# Exosomal miR-21 from tubular cells contributes to renal fibrosis by activating fibroblasts via targeting PTEN in obstructed kidneys

**DOI:** 10.7150/thno.62820

**Published:** 2021-08-02

**Authors:** Sheng Zhao, Wei Li, Weimin Yu, Ting Rao, Haoyong Li, Yuan Ruan, Run Yuan, Chenglong Li, Jinzhuo Ning, Siqi Li, Wu Chen, Fan Cheng, Xiangjun Zhou

**Affiliations:** 1 Department of Urology, Renmin Hospital of Wuhan University, Wuhan, 430060, China; 2 Department of Anesthesiology, Renmin Hospital of Wuhan University, Wuhan, 430060, China

**Keywords:** renal fibrosis, unilateral ureteral obstruction, exosome, microRNA

## Abstract

**Rationale:** Ureteral obstruction-induced hydronephrosis is associated with renal fibrosis and progressive chronic kidney disease (CKD). Exosome-mediated cell-cell communication has been suggested to be involved in various diseases, including renal fibrosis. However, little is known regarding how exosomes regulate renal fibrosis in obstructed kidneys.

**Methods:** We first examined the secretion of exosomes in UUO (unilateral ureteral obstruction) mouse kidneys and TGF-β1-stimulated tubular epithelial cells (NRK-52E). Exosomes from NRK-52E cells were subsequently harvested and incubated with fibroblasts (NRK-49F) or injected into UUO mice via the tail vein. We next constructed Rab27a knockout mice to further confirm the role of exosome-mediated epithelial-fibroblast communication relevant to renal fibrosis in UUO mice. High-throughput miRNA sequencing was performed to detect the miRNA profiles of TGFβ1-Exos. The roles of candidate miRNAs, their target genes and relevant pathways were predicted and assessed *in vitro* and *in vivo* by setting specific miRNA mimic, miRNA inhibitor, siRNA or miRNA LNA groups.

**Results:** Increased renal fibrosis was associated with prolonged UUO days, and the secretion of exosomes was markedly increased in UUO kidneys and TGF-β1-stimulated NRK-52E cells. Purified exosomes from TGF-β1-stimulated NRK-52E cells could activate fibroblasts and aggravate renal fibrosis *in vitro* and *in vivo*. In addition, the inhibition of exosome secretion by Rab27a knockout or GW4869 treatment abolished fibroblast activation and ameliorated renal fibrosis. Exosomal miR-21 was significantly increased in TGFβ1-Exos compared with Ctrl-Exos, and PTEN is a certain target of miR-21. The promotion or inhibition of epithelial exosomal miR-21 correspondingly accelerated or abolished fibroblast activation *in vitro*, and renal fibrosis after UUO was alleviated by miR-21-deficient exosomes *in vivo* through the PTEN/Akt pathway.

**Conclusion:** Our findings reveal that exosomal miR-21 from tubular epithelial cells may accelerate the development of renal fibrosis by activating fibroblasts via the miR-21/PTEN/Akt pathway in obstructed kidneys.

## Introduction

Hydronephrosis is a common clinical kidney disease that is usually caused by ureteral obstruction due to stones or tumors 1. Prolonged hydronephrosis is associated with renal fibrosis and progressive chronic kidney disease (CKD) 2. The pathological mechanism of renal fibrosis caused by hydronephrosis is complex and involves numerous cells, molecules and signaling pathways 3,4. The tubular epithelium is believed to be the primary target and epicenter of kidney injury 5. Ureteral obstruction leads to increased pressure in the renal pelvis and damage to tubular epithelial cells, which in turn triggers a series of cellular responses by interacting with interstitial cells to induce inflammatory cell infiltration and fibroblast activation 6-7. Emerging data indicate that extracellular vesicles, such as exosomes and microvesicles, play a fundamental role in cell-cell communication by transferring packages of information 8.

Exosomes are extracellular vesicles with a diameter of 30-150 nm and composed of lipids, proteins, and nucleic acids, including mRNAs, miRNAs or long nocoding RNAs 9. According to current studies, exosomes can be produced by almost all cell types under physiological and pathological conditions 10-12. Exosome secretion, which involves the transportation of multivesicular bodies, is regulated by Rab guanosine triphosphatases, including Rab27a and Rab27b 13. By shuttling abundant materials among cells, exosomes can deliver genetic information and signals, mediate intercellular communication and modulate cell behavior after being taken up by recipient cells 14.

MiRNAs are small noncoding RNAs that play a negative regulatory role at the posttranscriptional gene level and have been proven to be involved in the development of renal fibrosis caused by hydronephrosis 15,16. On the other hand, exosomes are also closely related to renal fibrosis caused by hydronephrosis, diabetes or ischemia reperfusion injury 17,18. However, most of these studies merely reflect the diagnostic and therapeutic value of exosomes 19-20. Although some recent studies have examined the biological role of exosomal miRNAs in the progression of renal fibrosis, they did not delve into the mechanism of exosome secretion or exosome mediated epithelial-fibroblast communication. 21-24.

In the present study, we demonstrated how tubular cell-derived exosomes are relevant to ureteral obstruction-induced renal fibrosis. Our results demonstrated the importance of tubular cell-derived exosomal miR-21 in the pathogenesis of ureteral obstruction-induced renal fibrosis and provide a novel avenue for developing therapeutics against the progression to CKD.

## Materials and methods

### Animal model

C57 male mice (20-25 g) were purchased from Hubei Provincial Centers for Disease Control and Prevention (Wuhan, China), and Rab27a knockout mice (weight 20-25 g) were purchased from Cyagen Biosciences (Guangzhou, China). UUO (unilateral ureteral obstructed) models were established by unilateral ureteral ligation according to previous studies. C57 mice were randomized into the sham group, UUO-3d group and UUO-7d group. Rab27a knockout mice were randomized into the sham group and UUO-7d group (n = 6 per group). The kidneys of mice were removed at 3 or 7 days after UUO, and kidney tissues were collected for various analyses.

To study the effects of exosomes, exosomes collected from NRK-52E cells treated with or without TGF-β1 were quantified and injected intravenously at UUO-1d, UUO-3d and UUO-5d (1 mg per mouse per time point). For miR-21-inhibited exosomes, anti-miR-21-LNA was previously transfected into NRK-52E cells.

**Figure 1:**
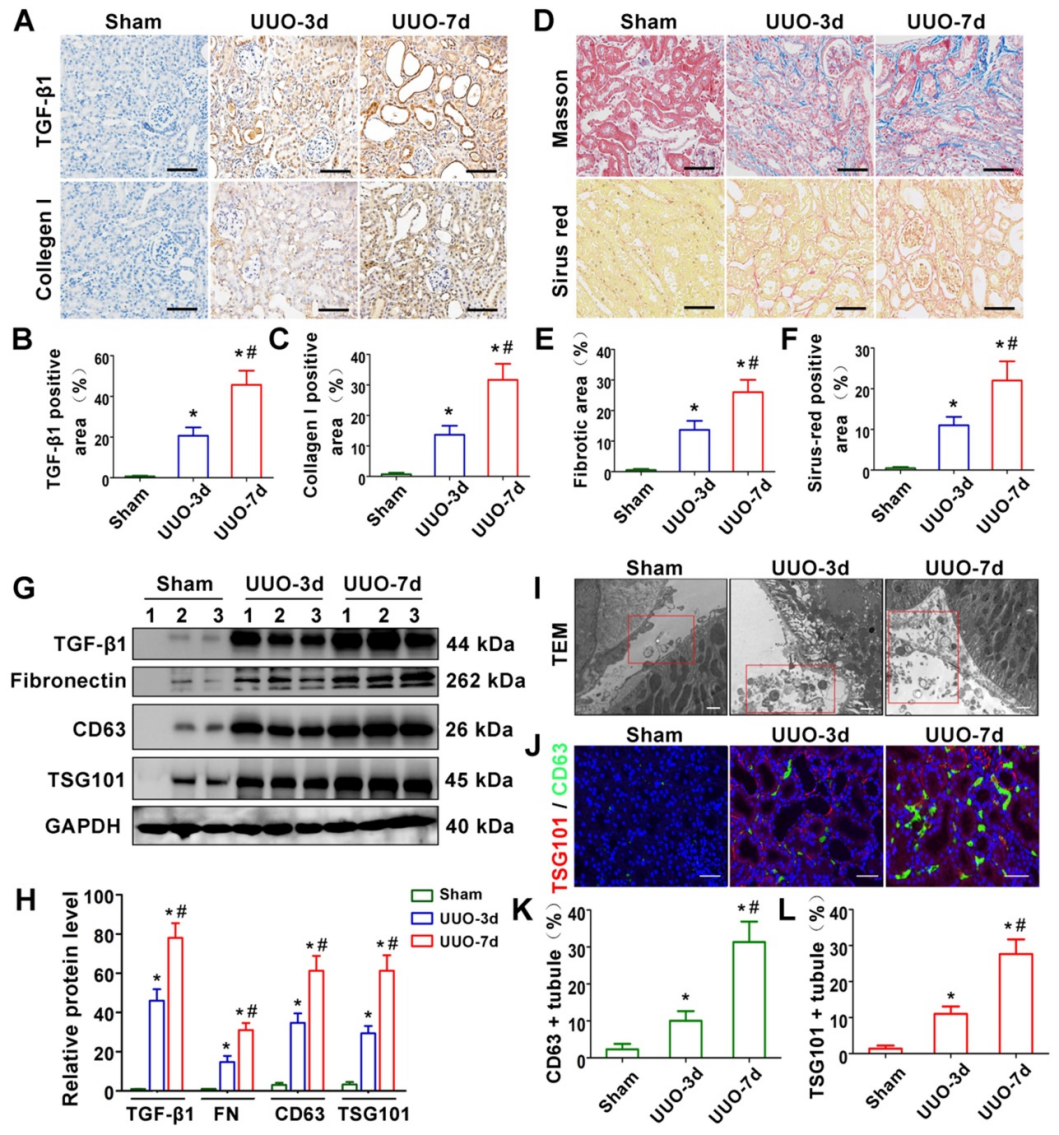
**TGF-β1 expression and exosome production are increased in UUO-induced renal fibrosis models. (A-C)** Representative micrographs of immunohistochemical staining **(A)** and quantitative data **(B,C)** show TGF-β1 and Collagen I expression at different time points after UUO. Scales bars = 50μm. ^*^p < 0.05 versus sham, ^#^p < 0.05 versus UUO-3d (n = 6). **(D-F)** Representative micrographs of Masson staining and Sirius red staining **(D)** and quantitative data **(E, F)** show collagen fiber accumulation after UUO. Scales bars=50 μm. ^*^p < 0.05 versus sham, ^#^p < 0.05 versus UUO-3d (n = 6). **(G, H)** Representative western blot **(G)** and quantitative data **(H)** on TGF-β1, fibronectin and exosomal-specific proteins CD63 and TSG101 after UUO. Numbers (1 to 3) indicate each individual animal in the given group. ^*^p < 0.05 versus sham, ^#^p < 0.05 versus UUO-3d (n = 6). **(I)** Transmission electron microscopy (TEM) shows the exosomes and microvesicles released by renal tubular epithelial cells after UUO. Scales bars = 300 nm. **(J)** Double immunofluorescence staining (green for CD63 and red for TSG101) demonstrates the generation of exosomes predominantly in tubular epithelial cells after UUO. Scales bars = 5 μm. **(K, L)** Quantitative determination of CD63- and TSG101-positive renal tubules. Data were obtained from 3 images per mouse, with 6 mice per group. ^*^p < 0.05 versus sham, ^#^p < 0.05 versus UUO-3d.

The experimental design is detailed in Figures 1, 3, 4 and 8. Animal protocols were approved by the Renmin Hospital of Wuhan University Animal Care and Use Committee. The methods were carried out in accordance with the approved guidelines.

### Cell culture and treatment

Rat renal tubular epithelial cells (NRK-52E) and rat fibroblasts (NRK-49F) were cultured with 10% fetal bovine serum and high-glucose Dulbecco's modified Eagle medium (DMEM) at 37 °C with 5% CO_2_ in a humidified environment. NRK-52E cells were treated with 5 ng/ml or 15 ng/ml TGF-β1 for 24 h to induce fibrotic changes followed by incubation in exosome-free medium. In some experiments, NRK-52E cells were pretreated with 10 μM GW4869 or transfected with miR-21 mimics, miR-21 inhibitor, their corresponding negative controls (NCs) or anti-miR-21-LNA (RiboBio, Guangzhou). NRK-52E cells at 60%-70% confluence were transfected using Lipofectamine 2000 according to the manufacturer's protocol. NRK-49F cells were pretreated with siPTEN (RiboBio, Guangzhou) or treated with exosomes (30 μg protein/mL) isolated from NRK-52E cells.

### Exosome isolation

Exosomes were extracted from conditioned media by different centrifugation procedures. Briefly, a supernatant sample was first transferred into a new 15 mL centrifuge tube for use, and after removing cell debris by centrifugation at 300 g for 10 min, 2000 g for 150 min, and 10000 g for 30 min, the supernatant was then ultracentrifuged at 100000 g for 2 h. All steps above were performed at 4 °C. The final pellets of exosomes were resuspended in approximately 100 μL of PBS and stored at 2-8 °C for 1 week and at -80 °C for long-time storage.

### Dynamic light scattering (DLS) and nanoparticle tracking analysis (NTA)

A ZetasizerNano ZS90 analysis system (Malvern Instruments, UK, Zetasizer version 7.12) was used to determine the size of the vesicles, and Nanosight (Merkel Technologies Ltd, Iserael, NTA version NTA 3.2 Dev Build 3.2.16) was used to characterize the concentration of exosomes as described previously.

### Transmission electron microscopy (TEM)

For TEM detection, mouse kidneys were first fixed with 2.5% glutaraldehyde and postfixed with 3% osmium tetroxide (OsO4) for 2 h. The samples were then dehydrated in a graded series of ethanol, embedded in Epon resin and sliced into 100 nm pieces. Final images were observed with a transmission electron microscope at 80 kV (Hitachi, Japan, H7500 TEM).

### High throughput miRNA sequencing

Purified exosomes from NRK-52E cells with or without TGF-β1 (15 ng/mL) stimulation separately underwent extraction of total RNA, including the small RNA fraction, and after extraction of the exosomal RNA, high-throughput sequencing was performed by Bioacme Biotechnology Company (Wuhan, China). Total RNA detection, gene library construction, and HiSeq/MiSeq sequencing were carried out according to the manufacturer's instructions.

### Fluorescence labeling of exosome

NRK-52E cells were first labeled with PKH-67 (a kind of fluorescent lipophilic cationic indocarbocyanine dye) for 2 h and washed three times with PBS. PKH-67-labeled exosomes from the conditioned media of NRK-52E cells were then incubated with NRK-49F cells for 24 h or injected into the mice via the tail vein, and exosome distribution was determined with immunofluorescence.

### Cell proliferation assays

The proliferation of NRK-49F cells was detected by Cell Counting Kit-8 (Nanjing Jiancheng Bioengineering Institute, Nanjing, China) according to the manufacturer's protocol. In addition, the expression of the proliferation-associated protein PCNA and fibroblast-specific protein Fsp-1 was detected by western blot or immunofluorescence, which can reflect the proliferation of fibroblasts.

### Western blot

The concentration of the protein from animals or cells was detected using the bicinchoninic acid method. Proteins in each group were subjected to 12% or 15% sodium dodecyl sulfate-polyacrylamide gel electrophoresis and transferred onto a polyvinylidene difluoride membrane for 1-2 h at 200 mA, according to their molecular weight. After blocking with Tris-buffered saline/Tween-20 containing 5% dried skim milk for 1 h at room temperature (approximately 25 °C), the membranes were incubated with primary antibodies overnight at 4 °C. The primary antibodies were as follows: TGF-β1 (Sigma, SAB4502954), CD63 (Affinity Biosciences, AF5117), TSG-101 (Abcam, ab125011), Fibronectin (Proteintech Group, 15613-1-AP), α-SMA (Boster Biological Technology, BM0002), Collagen I (Novus Biologicals, NB600-408), E-cadherin (Abcam, ab76319), Fsp-1 (Cell Signaling Technology, 13018), PCNA (Abcam, ab92552), PTEN (Cell Signaling Technology, 9559), and p-Akt (Abcam, ab18785). Membranes were subsequently incubated for 1 h at room temperature with anti-mouse/rabbit secondary antibody (P/N 925-32210 or P/N 925-32211; LI-COR Biosciences), which was conjugated to IRDye 800CW. Finally, the fluorescence signal emitted by the secondary antibody was quantified by a western blot detection system (Odyssey Infrared Imaging; LI-COR Biosciences), and semiquantitative analysis was conducted to determine the corresponding protein expression levels (Image Studio version 5.2.5; LI-COR Biosciences).

### qRT-PCR analysis

Total RNA was extracted from cells using TRIzol Reagent. For mRNA detection, RNase-free water (100 μL) was added to dissolve the extracted total RNA. First, cDNA was synthesized by an EntiLink™ First-strand cDNA Synthesis Kit. Real-time fluorescent quantitative PCR was performed based on the instructions of the StepOne™ Real-Time PCR Kit. Three complex wells were made in each sample by real-time PCR using the EnTurbo™ SYBR Green PCR SuperMix Kit. The PCR primers were as follows: PTEN: 5′-CACCAGTTCGTCCCTTTCCA-3′ (forward), 5′-TGACAATCATG TTGCAGCAATTC-3′ (reverse); GAPDH: 5′-TCCATGACAACTTTGGCATTG-3′ (forward), 5′-TCA CGCCACAGCTTTCCA-3′ (reverse). For miRNA analysis, exosomal miRNAs were isolated with a SeraMir Exosome RNA Purification Kit (System Biosciences, Mountain View, USA). qRT-PCR was performed using FastStart Universal SYBR Green Master Mix (Roche, Indianapolis, USA) with the miRNA-specific forward primer and the universal reverse primer (RiboBio, Guangzhou, China). The small nuclear RNA U6 was used as an internal control to normalize the expression of miR-21.

### Luciferase reporter assay

The PTEN 3'UTR carrying a miR-21 binding site was constructed by PCR and subsequently cloned into the pMIR-REPORT vector to construct the wild-type PTEN luciferase reporter. The wild-type PTEN construct and miR-21 mimic or scrambled sequence oligonucleotides were co-transfected into NRK-52E cells using Lipofectamine 2000. Lysates were harvested after 36 h, and luciferase activity was measured using the dual-luciferase assay (Promega, Madison, WI, USA).

### Histology, immunohistochemical and immunofluorescence staining

Paraffin-embedded mouse kidney sections were prepared by a routine procedure. Masson's trichrome staining, Sirius red staining, immunohistochemical staining and immunofluorescence staining were performed according to routine protocols.

### Statistical analyses

All the results are expressed as the mean ± standard deviation. Statistical analysis of the data was performed using SPSS 17.0 software. Comparisons between groups were made using one-way ANOVA, followed by Student-Newman-Kuels test. p < 0.05 was considered to represent a significant difference.

## Results

### TGF-β1 expression and exosome production are increased in UUO-induced renal fibrosis models

We first evaluated renal fibrosis and TGF-β1 expression in UUO-3d and UUO-7d models by Masson's trichrome, Sirius red staining, western blotting and immunohistochemistry (Figure 1A-H). The results indicated that TGF-β1 expression and renal fibrosis were positively correlated with the days of obstruction. To further clarify whether exosomes play a role in UUO models, transmission electron microscopy, immunofluorescence and western blotting were conducted. Transmission electron microscopy showed a significant increase in extracellular vesicle secretion after UUO. Moreover, many extracellular vesicles had an exosome diameter of 30-150 nm (Figure 1I). Western blot (Figure 1G and H) and immunostaining (Figure 1J-L) for CD63 and TSG101 (exosomal markers) revealed that exosomes were apparently increased in the UUO-3d group and further increased in the UUO-7d group. In addition, exosomes were predominantly distributed around the renal tubular epithelium (Figure 1J).

### TGF-β1 promotes the secretion of exosomes by renal tubular epithelial cells and activates fibroblasts *in vitro*

**Figure 2:**
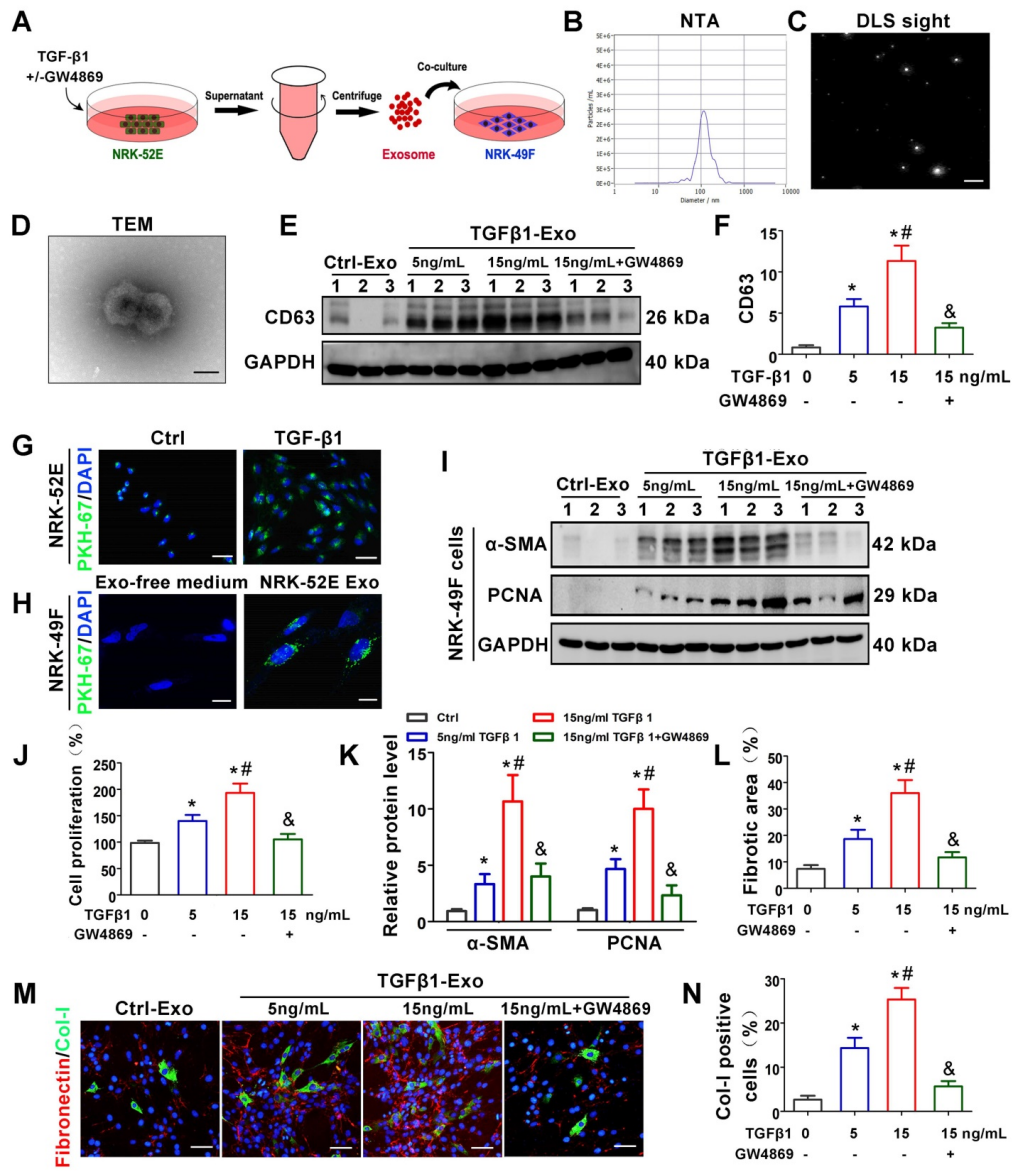
**TGF-β1 promotes the secretion of exosomes by renal tubular epithelial cells and activates fibroblasts *in vitro*. (A)** Schematic diagram of experimental process. Exosomes from NRK-52E cells treated without (Ctrl-Exos) or with TGF-β1 (TGFβ1-Exos) were extracted and incubated with NRK-49F cells.** (B,C)** DLS and NTA of exosomes from NRK-52E cells.** (D)** TEM image of exosomes isolated from NRK-52E cells. Scale bar = 100 nm.** (E, F)** Representative western blot **(E)** and quantitative data **(F)** of CD63 as an exosome marker in exosomes from TGF-β1- or GW4869-treated NRK-52E cells. Numbers (1 to 3) indicate each independent treatment in the given group. ^*^p < 0.05 versus Ctrl-Exos, ^#^p < 0.05 versus 5 ng/ml TGFβ1-Exos, ^&^p < 0.05 versus 15 ng/ml TGFβ1-Exos (n = 3). **(G)** Fluorescent staining image of PKH-67-labeled NRK-52E cells. Scales bars=50 μm. **(H)** Fluorescent staining image of NRK-52E cell-derived exosomes taken up by NRK-49F cells. Scales bars=10 μm. **(I, K)** Representative western blot **(I)** and quantitative data **(K)** of α-SMA and PCNA in NRK-49F cells incubated with exosomes from TGF-β1- or GW4869-treated NRK-52E cells. Numbers (1 to 3) indicate each independent treatment in the giving group. ^*^p < 0.05 versus Ctrl-Exos, ^#^p < 0.05 versus 5 ng/ml TGFβ1-Exos, ^&^p < 0.05 versus 15 ng/ml TGFβ1-Exos (n = 3). **(J)** Proliferation rate of NRK-49F cells incubated with NRK-52E cell-derived exosomes measured by CCK-8. ^*^p < 0.05 versus Ctrl-Exos, ^#^p < 0.05 versus 5 ng/ml TGFβ1-Exos, ^&^p < 0.05 versus 15 ng/mL TGFβ1-Exos (n = 3). **(L-N)** Double immunofluorescence staining (green for Col-I and red for fibronectin) demonstrates the expression of Col-I and fibronectin in NRK-49F cells incubated with NRK-52E cell-derived exosomes. Scales bars=50 μm. ^*^p < 0.05 versus Ctrl-Exos, ^#^p < 0.05 versus 5 ng/ml TGFβ1-Exos, ^&^p < 0.05 versus 15 ng/mL TGFβ1-Exos.

We next investigated the potential role of tubule-derived exosomes in mediating fibroblast activation. As a master fibrogenic factor, TGF-β1 was used to stimulate the condition of injured/stressed tubular cells in UUO kidneys. After 24 h of incubation with different concentrations of TGF-β1 with or without GW4869 (exosome secretion inhibitor), NRK-52E cells were incubated with exosome-free medium for another 24 h, and exosomes from conditioned media were collected to stimulate normal rat kidney interstitial fibroblasts (NRK-49F cells) (Figure 2A). As shown in Figure 2B-D, NTA, DLS and TEM displayed the majority of isolated extracellular vesicles being exosomes, as defined by their sizes. Western blot analysis of CD63 confirmed that exosome secretion was dependent on the concentration of TGF-β1 and that GW4869 treatment obviously inhibited extracellular matrix deposition (Figure 2E-F). We next labeled NRK-52E cells with PKH-67, a lipophilic fluorescent dye for tracing, and the significantly enhanced fluorescence intensity further confirmed the increase in exosome secretion after TGF-β1 treatment (Figure 2G). In addition, PKH-67-labeled exosomes incubated with NRK-49F cells were taken up by fibroblasts (Figure 2H). Furthermore, exosomes isolated from TGF-β1-treated NRK-52E cells were able to induce NRK-49F cell proliferation and matrix production, as manifested by increased expression of α-SMA, PCNA, Col-I and fibronectin. However, GW4869 treatment apparently reversed the above changes (Figure 2I-N).

### Tubular cell-derived exosomes promote renal fibrosis *in vivo*

**Figure 3:**
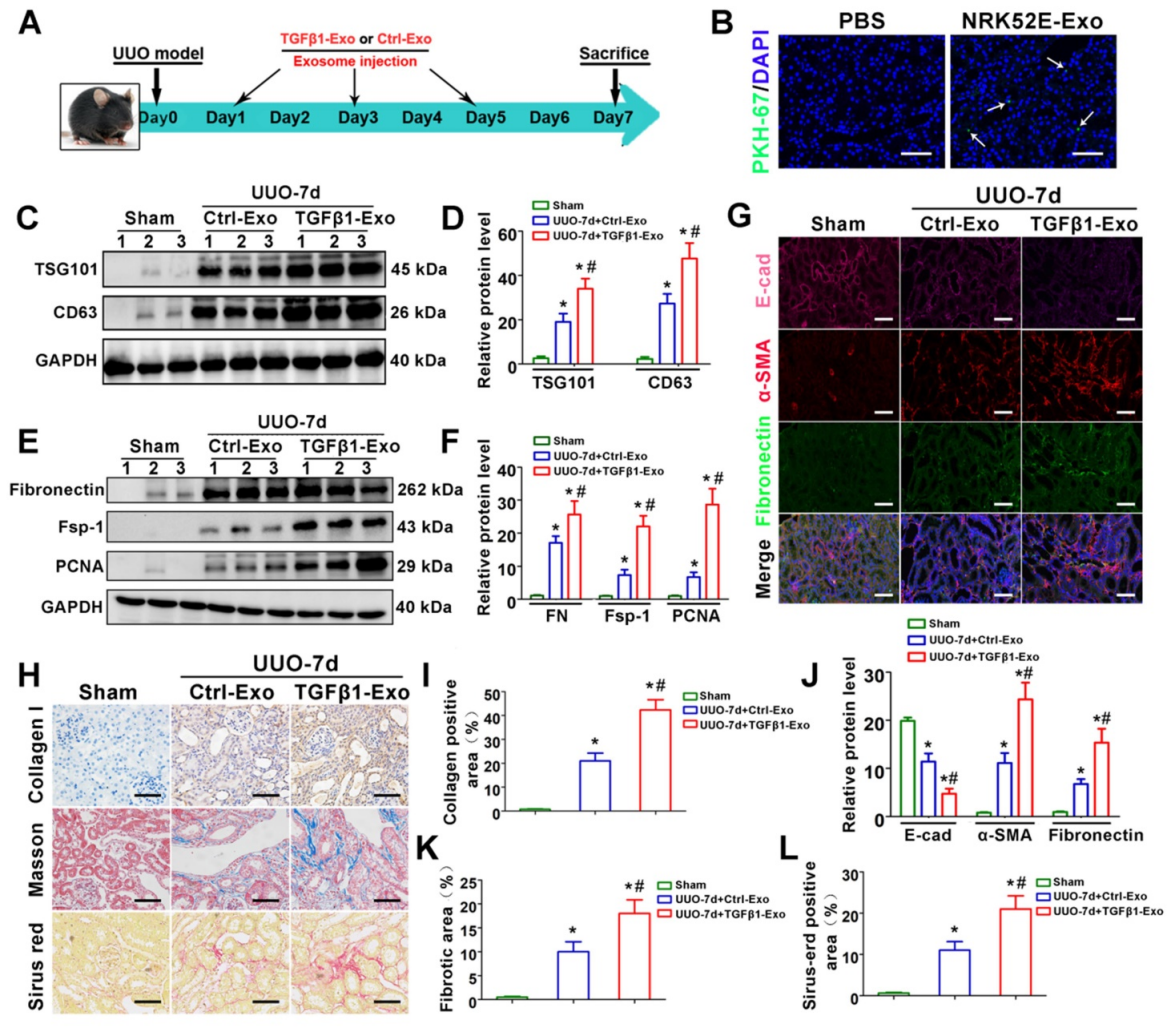
** Tubular cell-derived exosomes promote renal fibrosis *in vivo*. (A)** Experimental design. TGFβ1-Exos or Ctrl-Exos from NRK-52E cells were injected into UUO mice by tail vein injection at 1, 3 and 5 days. **(B)** Representative images show the presence of PKH-67-labeled TGFβ1-Exos isolated from NRK-52E cells in mouse kidneys after intravenous injection. Kidney sections were examined at 24 h after injection. Arrows indicate PKH-67 labeled exosomes (green). Scales bars=50 μm. **(C, D)** Representative western blot **(C)** and quantitative data **(D)** of TSG101, CD63 and fibronectin in UUO kidneys injected with Ctrl-Exos or TGFβ1-Exos. Numbers (1 to 3) indicate each individual animal in the given group. ^*^p < 0.05 versus sham, ^#^p < 0.05 versus UUO7d+Ctrl-Exo (n = 6). **(E, F)** Representative western blot **(E)** and quantitative data **(F)** of fibronectin, Fsp-1 and PCNA in UUO kidneys injected with Ctrl-Exos or TGFβ1-Exos. Numbers (1 to 3) indicate each individual animal in the given group. ^*^p < 0.05 versus sham, ^#^p < 0.05 versus UUO7d+Ctrl-Exo (n = 6). **(G, J)** Treble immunofluorescence staining** (G)** and quantitative data **(J)** demonstrate the expression of E-cad, α-SMA and fibronectin in UUO kidneys injected with Ctrl-Exos or TGFβ1-Exos. Scales bars=50μm. ^*^p < 0.05 versus sham, ^#^p < 0.05 versus UUO7d+Ctrl-Exo. **(H, I, K, L)** Representative micrographs of Col-I immunohistochemical staining, Masson staining, Sirius red staining **(H)** and quantitative data **(I, K, L)** show fibronectin and collagen deposition in UUO kidneys injected with Ctrl-Exos or TGFβ1-Exos. Scales bars=50 μm. ^*^p < 0.05 versus sham, ^#^p < 0.05 versus UUO7d+Ctrl-Exo.

To investigate the *in vivo* relevance of tubule-derived exosomes to UUO-induced fibrosis, we carried out animal studies using a UUO model by injecting TGFβ1-Exos isolated from TGF-β1-treated NRK-52E cells. The experimental design is shown in Figure 3A. UUO mice were injected with Ctrl-Exo or TGFβ1-Exo from the same amount of NRK-52E cells at 1, 3 and 5 days. We first examined whether exosomes could be delivered into kidneys after tail vein injection. As shown in Figure 3B, PKH-67-labeled TGFβ1-Exos from NRK-52E cells were observed in obstructed kidneys *in vivo*. Moreover, injection of TGFβ1-Exo unsurprisingly induced higher expression of CD63 and TSG101 than Ctrl-Exo injection (Figure 3C-D). We then detected the effects of exosome injection on UUO kidneys. As shown in Figure 3E-L, immunostaining for Col-I, E-cad, α-SMA and fibronectin and western blot analysis of fibronectin, Fsp-1 and PCNA or Sirius red staining showed that TGFβ1-Exos promoted the proliferation of fibroblasts and aggravated fibronectin and collagen deposition in obstructed kidneys at 7 days after UUO compared to Ctrl-Exos.

### Rab27a knockout inhibits exosome secretion and alleviates UUO-induced renal fibrosis *in vivo*

**Figure 4:**
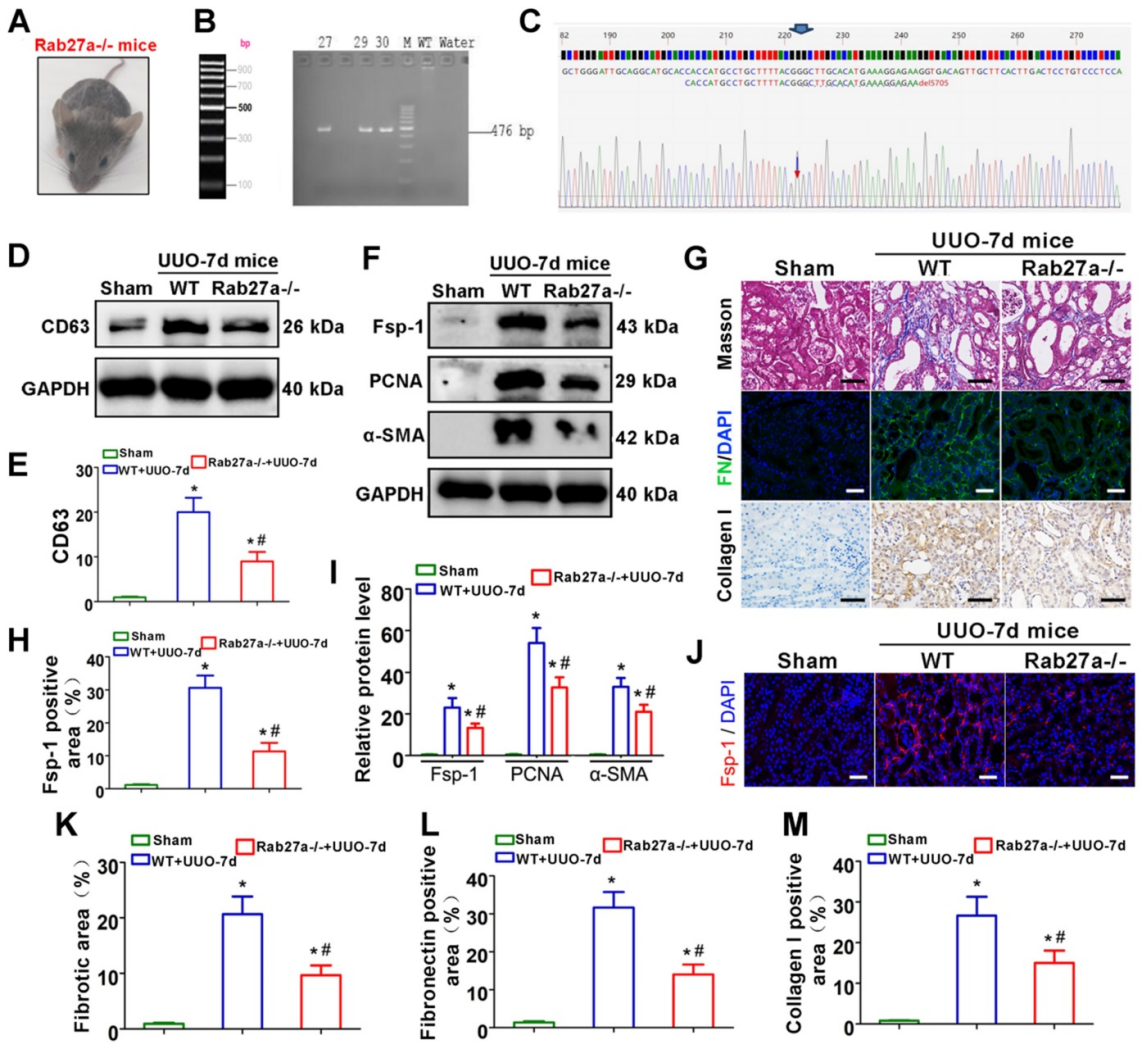
** Rab27a knockout inhibits exosome secretion and alleviates UUO-induced renal fibrosis *in vivo*. (A)** Rab27a^-/-^ mouse. **(B, C)** Rab27a knockout was confirmed by PCR screening (targeted allele: 476 bp) **(B)** and sequencing confirmation **(C)**. **(D, E)** Representative western blot **(D)** and quantitative data **(E)** of CD63 in Rab27a knockout kidneys after UUO (n = 6). ^*^p < 0.05 versus sham, ^#^p < 0.05 versus WT+UUO-7d.** (F, I)** Representative western blot **(F)** and quantitative data **(I)** of Fsp-1, PCNA and α-SMA in Rab27a knockout kidneys after UUO (n = 6). ^*^p < 0.05 versus sham, ^#^p < 0.05 versus WT+UUO-7d.** (H, J)** Representative immunofluorescence micrographs **(J)** and quantitative data **(H)** show Fsp-1 expression (n = 6). Scales bars=50 μm. ^*^p < 0.05 versus sham, ^#^p < 0.05 versus WT+UUO-7d.** (G, K-M)** Masson staining, Col-I immunohistochemical staining and fibronectin immunofluorescence staining indicated the deposition of collagens and fibronectin. Representative micrographs **(G)** and quantitative data **(K-M)** are presented (n = 6). Scales bars=50 μm. ^*^p < 0.05 versus sham, ^#^p < 0.05 versus WT+UUO-7d.

As shown in Figures 4A-C, Rab27a knockout mice (Rab27a^-/-^) were successfully constructed and confirmed. CD63 expression was then detected by western blot, which indicated that Rab27a knockout reduced exosome secretion in UUO kidneys (Figure 4D-E). We next examined the activation of fibroblasts and the deposition of fibronectin and collagens. The results indicated that Rab27a knockout obviously suppressed the activation of fibroblasts, which was confirmed by detecting the expression of α-SMA, PCNA and Fsp-1 by western blotting (Figure 4F, I) and immunofluorescence (Figure 4F, H, J). Meanwhile, Masson staining, Col-I immunohistochemical staining and fibronectin immunofluorescence staining confirmed that Rab27a knockout ameliorated renal fibrosis after UUO by inhibiting collagen and fibronectin deposition (Figure 4G, K-M).

### Tubule cell-derived exosomal miR-21 promotes fibroblast activation *in vitro*

**Figure 5:**
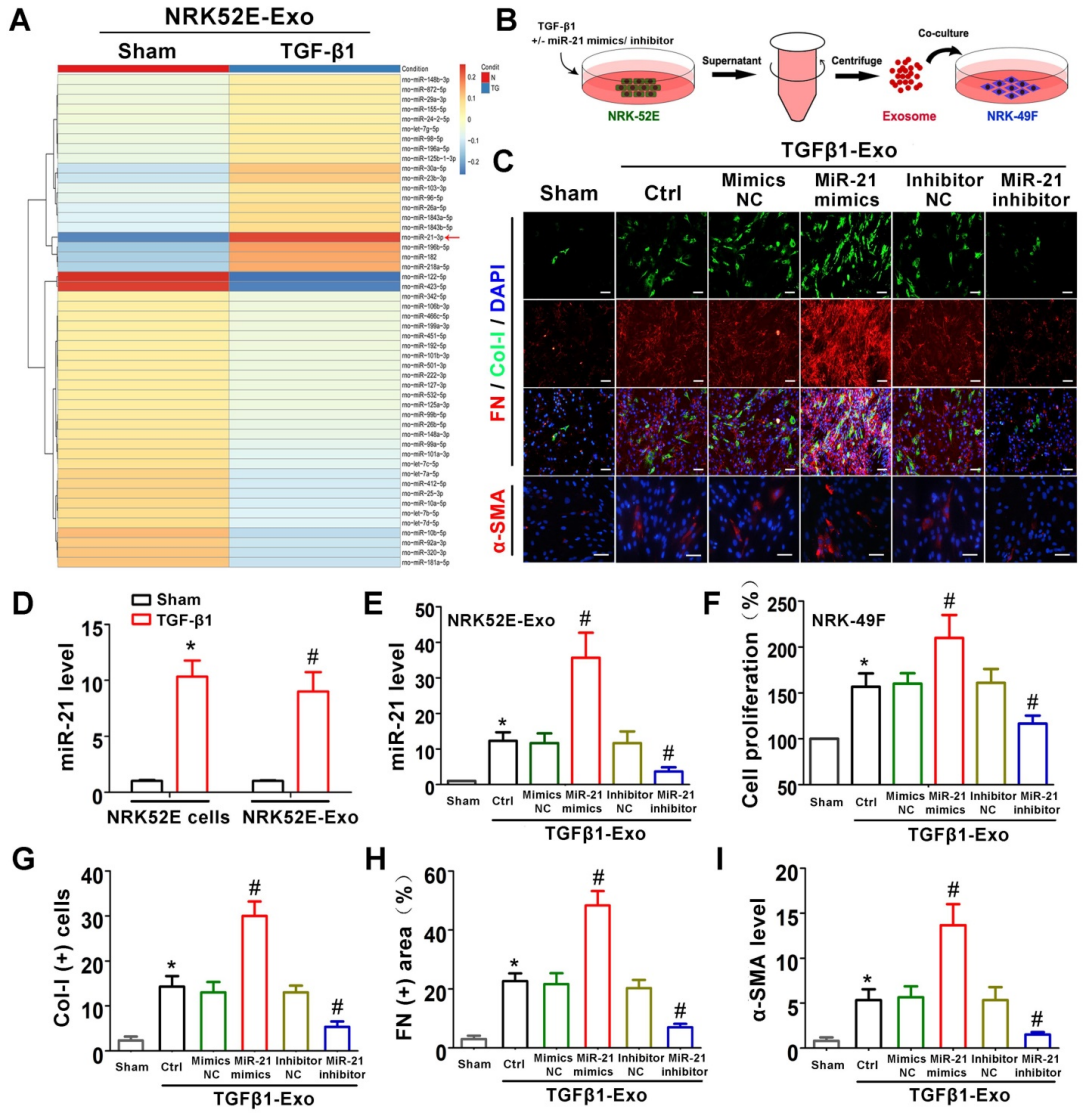
** Tubule cell-derived exosomal miR-21 promotes fibroblast activation *in vitro*. (A)** NRK-52E-delivered exosomal miRNA sequencing after TGF-β1 (15 ng/ml) treatment. **(B)** Experimental design. Concentration of TGF-β1, 15 ng/ml. **(C, G-I)** Representative immunofluorescence micrographs **(C)** and quantitative data **(G-I)** show Col-I, fibronectin and α-SMA expression in NRK-49F cells after stimulation with NRK-52E-delivered exosomes. Scales bars=50 μm. ^*^p < 0.05 versus sham, ^#^p < 0.05 versus Ctrl. **(D)** PCR detection of miR-21 in exosomes and NRK-52E cells after TGF-β1 treatment (n = 3). ^*^p < 0.05 versus sham in cells, ^#^p < 0.05 versus sham in exosomes. **(E)** PCR detection of miR-21 in NRK-52E-delivered exosomes after miR-21 mimic or inhibitor transfection (n = 3). ^*^p < 0.05 versus sham, ^#^p < 0.05 versus Ctrl. **(F)** Proliferation of NRK-49F cells after stimulation with exosomes from NRK-52E cells (n = 3). ^*^p < 0.05 versus sham, ^#^p < 0.05 versus Ctrl.

The above animal and cell experiments have demonstrated the role of tubule cell-delivered exosomes in promoting fibroblast activation in UUO models; however, the specific molecular mechanism remains unknown. To explore whether tubular cell-derived exosomal miRNAs play a role, miRNA sequencing was conducted (Figure 5A). Among all altered miRNAs, miR-21 was the most significantly elevated, and we further confirmed that miR-21 was induced in NRK-52E cells and NRK-52E-delivered exosomes after TGF-β1 treatment by PCR (Figure 5D). To confirm the role of exosomal miR-21, miR-21 mimics or inhibitor was transfected into NRK-52E cells to overexpress or inhibit exosomal miR-21 (Figure 5B), and the transfection effect was verified to be effective compared to the corresponding NC group by PCR (Figure 5E). Immediately after cell transfection and TGF-β1 treatment, corresponding exosomes were added to NRK-49F cells. As shown in Figure 5C and Figure 5F-I, the expression of Col-I, fibronectin and α-SMA in NRK-49F cells as well as cell proliferation apparently increased when stimulated with exosomes from miR-21 mimic-transfected NRK-52E cells. Similarly, the expression of Col-I, fibronectin and α-SMA in NRK-49F cells as well as cell proliferation apparently decreased when stimulated with exosomes from miR-21 inhibitor-transfected NRK-52E cells. However, mimic NC or inhibitor NC transfection showed no significant changes.

### PTEN is a potential target of miR-21

**Figure 6:**
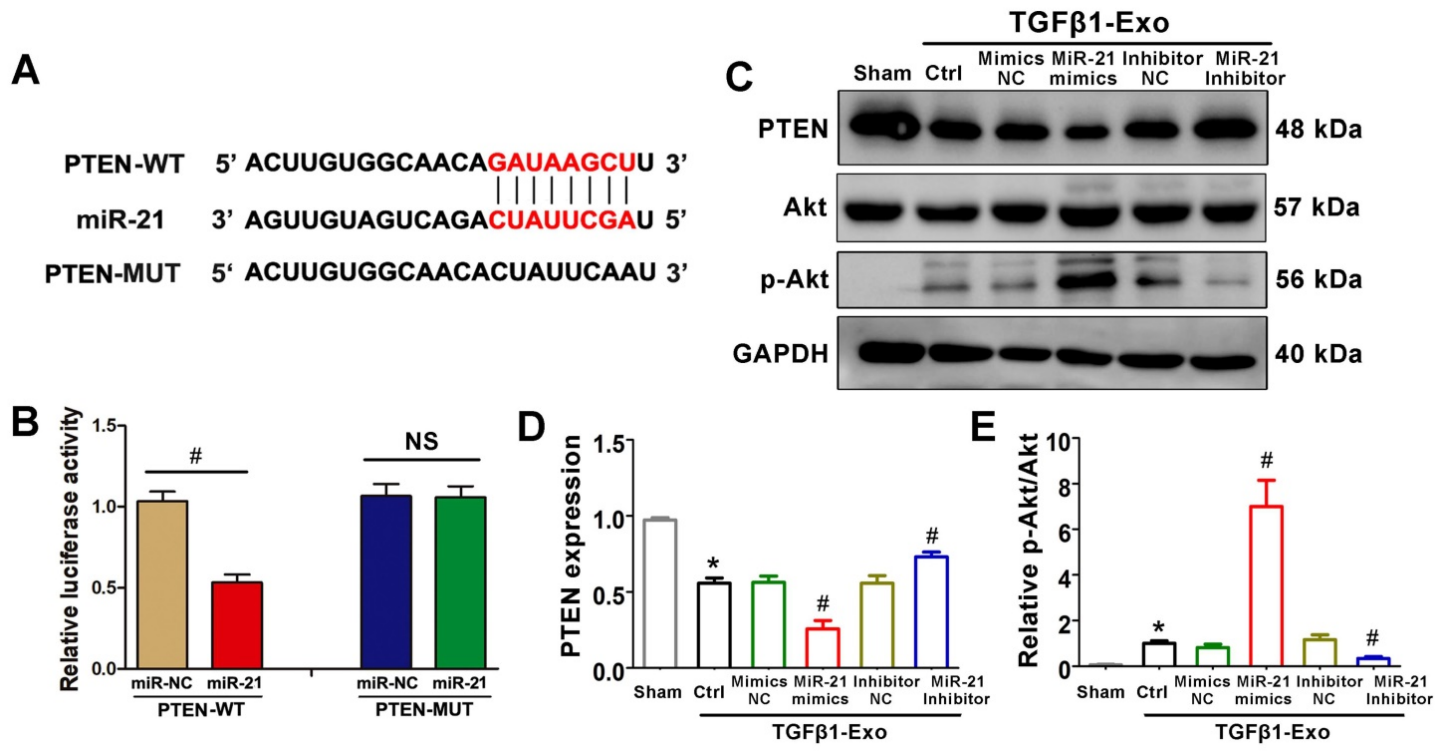
** PTEN is a potential target of miR-21. (A)** Predicted potential miR-21 binding site in the 3'-UTR of PTEN mRNA. **(B)** Luciferase activity in NRK-49F cells transfected with negative control or miR-21 mimic together with reporter vector containing PTEN-mut binding sequences. ^#^p < 0.05 versus miR-NC. NS, no significant difference. **(C-E)** Representative western blot **(C)** and quantitative data **(D, E)** show PTEN, Akt and p-Akt expression in NRK-49F cells after being treated with NRK-52E-delivered exosomes (n = 3). ^*^p < 0.05 versus sham, ^#^p < 0.05 versus Ctrl.

We further investigated the possible mechanism by which tubular cell-derived exosomal miR-21 mediates fibroblast activation. TargetScan software predicted that the 3′-untranslated region (UTR) of the PTEN gene was the conserved binding site of miR-21 (Figure 6A). To verify the relationship between miR-21 and PTEN, a wild-type PTEN (PTEN-WT) luciferase reporter construct carrying the PTEN 3′UTR was constructed. After the transfection of a miR-21 mimic with PTEN-WT into NRK-49F cells, miR-21 mimics suppressed the luciferase activity in PTEN-miR-21-transfected cells compared to the Ctrl group (Figure 6B). Finally, we confirmed the expression levels of PTEN and downstream p-Akt in NRK-49F cells by western blot. Unsurprisingly, PTEN was apparently decreased and p-Akt was increased when stimulated with exosomes from miR-21 mimic-transfected NRK-52E cells, but these changes were mitigated when stimulated with exosomes from miR-21 inhibitor-transfected NRK-52E cells (Figure 6C-E).

### Tubule cell-derived exosomal miR-21 mediates fibroblast activation through the PTEN/Akt pathway *in vitro*

**Figure 7:**
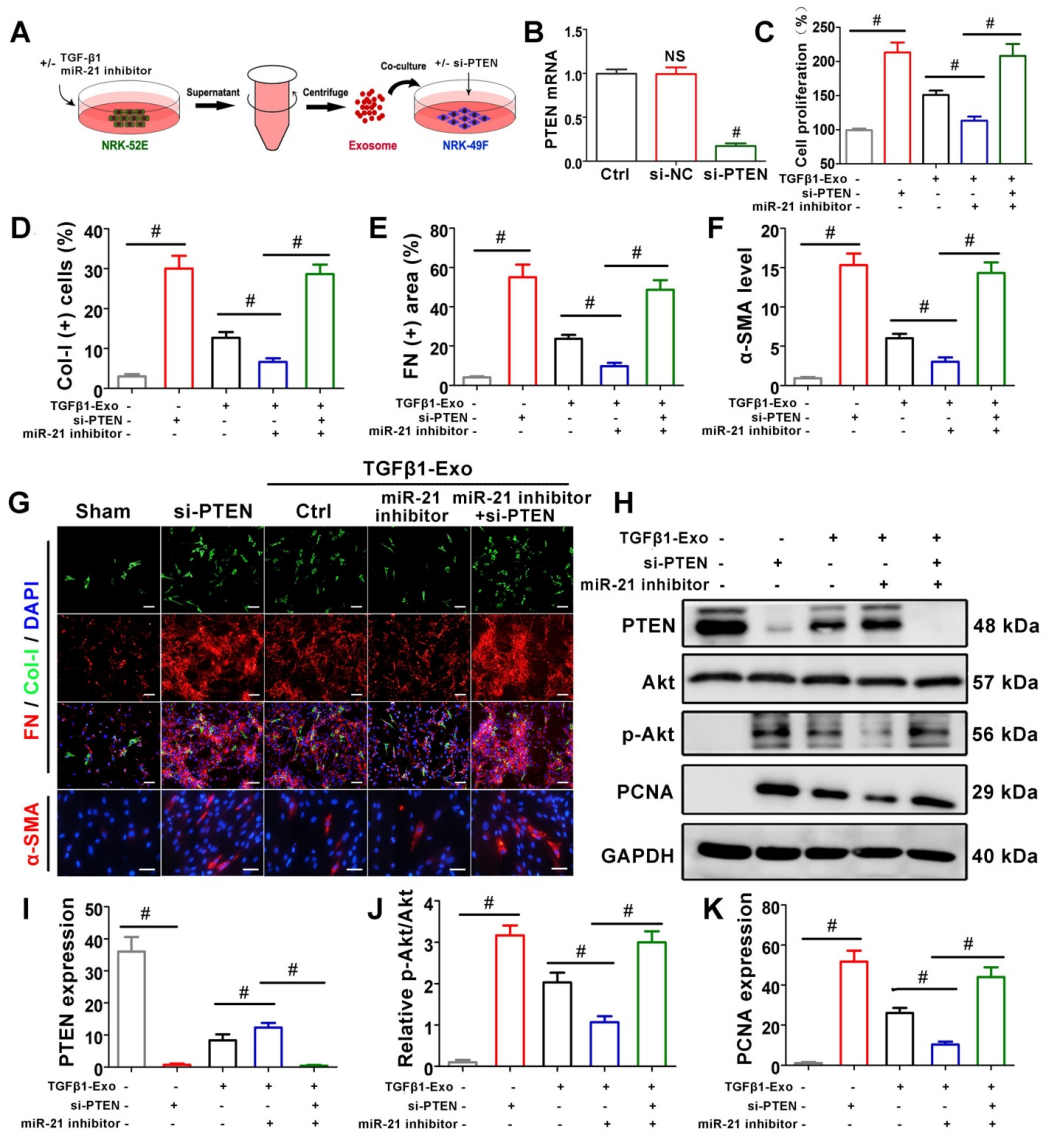
** Tubule cell-derived exosomal miR-21 mediates fibroblast activation through the PTEN/AKT pathway *in vitro*. (A)** Experimental design. Concentration of TGF-β1, 15 ng/ml. **(B)** PTEN mRNA level after siPTEN treatment. NS, no significant difference versus Ctrl, ^#^p < 0.05 versus Ctrl. **(C)** Proliferation of NRK-49F cells after miR-21 inhibitor or siPTEN transfection (n = 3). ^#^p < 0.05. **(D-G)** Representative immunofluorescence micrographs **(G)** and quantitative data **(D-F)** show Col-I, fibronectin and α-SMA expression in NRK-49F cells after miR-21 inhibitor or siPTEN transfection (n = 3). Scales bars=50 μm. ^#^p < 0.05. **(H-K)** Representative western blot **(H)** and quantitative data **(I-K)** show PTEN, Akt, p-Akt and PCNA expression in NRK-49F cells after miR-21 inhibitor or siPTEN transfection (n = 3). ^#^p < 0.05.

To further confirm the role of miR-21 in PTEN, NRK-49F cells were treated with PTEN siRNA before receiving NRK-52E-delivered exosomes (Figure 7A), and PTEN mRNA was significantly suppressed compared with the si-NC group (Figure 7B). CCK-8 (Figure 7C) and PTEN expression by western blot (Figure 7H, K) showed that siPTEN transfection without exosome intervention significantly promoted NRK-49F proliferation. In the exosome intervention groups, miR-21 inhibitor transfection alone inhibited NRK-49F proliferation but was apparently reversed after simultaneous transfection with siPTEN. Immunofluorescence staining showed that the expression of Col-I, fibronectin and α-SMA in NRK-49F cells apparently increased after siPTEN transfection. In the exosome intervention groups, miR-21 inhibitor transfection alone inhibited the above indicators but was apparently reversed after simultaneous transfection with siPTEN. Pathway-associated proteins PTEN and p-Akt were then detected by western blot, as shown in Figure 7H-J. siPTEN transfection without exosome intervention significantly suppressed PTEN but accelerated p-Akt. In the exosome intervention groups, miR-21 inhibitor transfection alone accelerated PTEN and suppressed p-Akt, but this effect was reversed after co-transfection with siPTEN. The results above indicated that tubule cell-derived exosomal miR-21 could mediate fibroblast activation through the PTEN/AKT pathway.

### Tubular cell-derived exosomal miR-21 promotes renal fibrosis through the PTEN/Akt pathway *in vivo*

**Figure 8:**
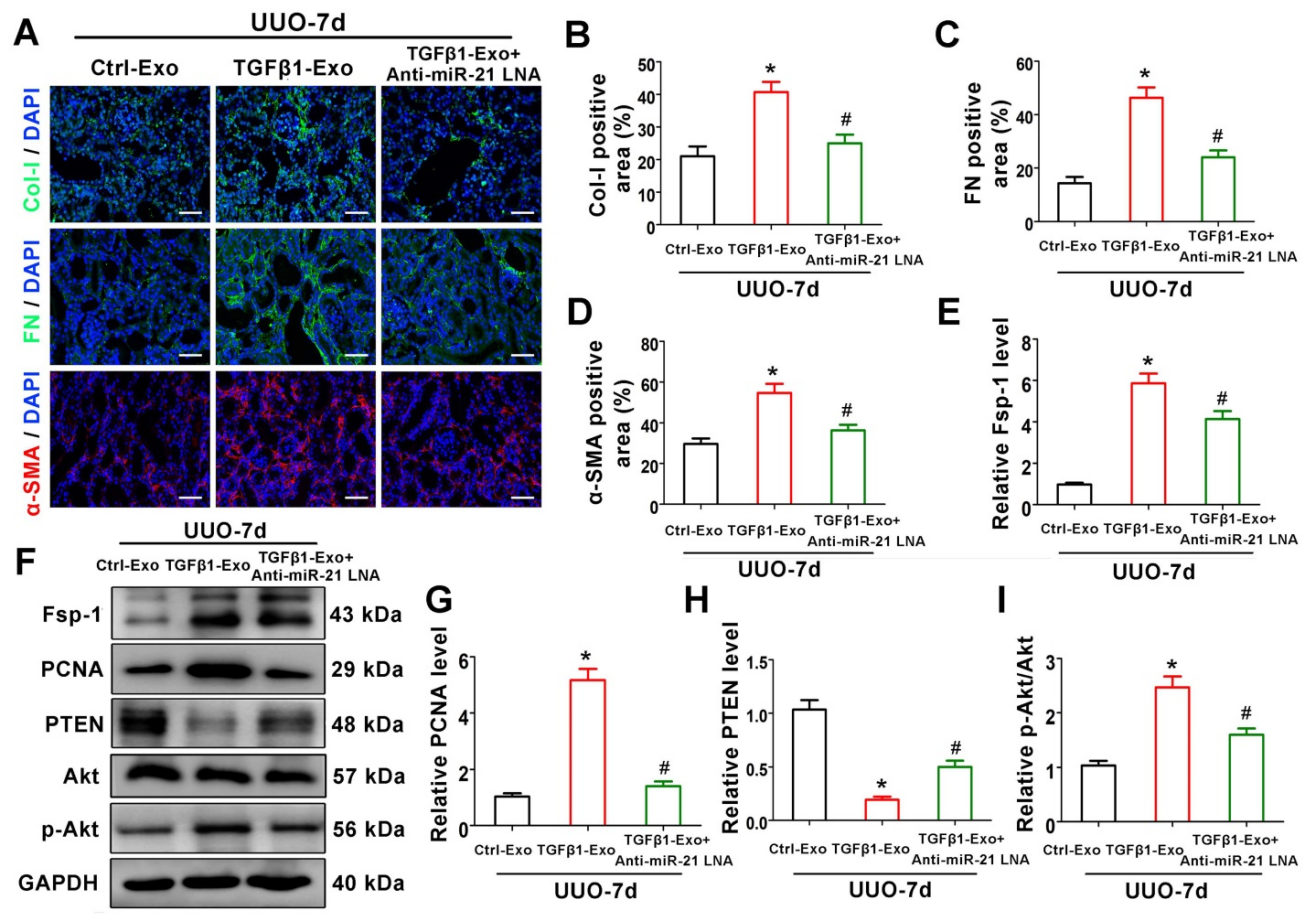
** Tubular cell-derived exosomal miR-21 promotes renal fibrosis through the PTEN/AKT pathway *in vivo*. (A-D)** Representative immunofluorescence micrographs **(A)** and quantitative data **(B-D)** show Col-I, fibronectin and α-SMA expression in mouse kidneys after intravenous exosome injection (n = 6). Scales bars=50 μm. ^*^p < 0.05 versus Ctrl-Exo, ^#^p < 0.05 versus TGFβ1-Exo. **(E-I)** Representative western blot **(F)** and quantitative data **(E, G-I)** show Fsp-1, PCNA, PTEN, Akt and p-Akt expression in mouse kidneys after intravenous exosome injection (n = 6). ^*^p < 0.05 versus Ctrl-Exo, ^#^p < 0.05 versus TGFβ1-Exo.

To verify the mechanism of exosomal miR-21 *in vivo*, animal studies were carried out using the UUO model by TGFβ1-Exo injection. Interestingly, TGFβ1-Exos from NRK-52E cells increased the extracellular matrix and fibroblast proliferation at 7 days after UUO, as determined by immunostaining of Col-I, fibronectin, and α-SMA and western blotting of Fsp-1 and PCNA, while inhibiting the expression of miR-21 in tubule cell-derived exosomes inhibited fibroblast activation and extracellular matrix production (Figure 8A-D, 8E-G). We further found that tubular cell-derived exosomes downregulated PTEN and activated p-Akt in kidneys following UUO. In addition, the inhibition of miR-21 in exosomes upregulated PTEN and inhibited p-Akt (Figure 7F, H-I).

## Discussion

The renal tubular epithelium is susceptible to various adverse impacts, such as ischemia, hydronephrosis or toxicological effects 25. Stressed tubular epithelial cells are believed to engage in multiple intercellular crosstalk with interstitial cells, especially fibroblasts, resulting in their activation and proliferation 26. Such epithelial-mesenchymal communication has become a new paradigm in renal fibrogenesis. In the present study, we found that exosomes were markedly facilitated in TGF-β1-stimulated renal tubular epithelial cells and that exosomes could remarkably activate fibroblasts and exacerbate renal fibrosis. Furthermore, blockade of exosome secretion by pharmacologic and genetic approaches inhibits fibroblast activation and renal fibrosis. In addition, we further proved that enriched exosomal miR-21 from injured renal tubular epithelial cells is one of the main molecules for the activation of fibroblasts via the miR-21/PTEN/Akt pathway in obstructed kidneys. Our findings provide novel insights into the pathological mechanisms by which exosomes indicate that epithelial-mesenchymal communication promotes fibrosis in obstructed kidneys.

Exosomes, with an average diameter of 30-150 nanometers, are a subset of extracellular vesicles (EVs). Their diverse constituents include nucleic acids, proteins, lipids and metabolites 9. Earlier studies have shown that the contents of exosomes are not only determined by their cellular origin but also related to cellular status and extracellular microenvironment 27. Although exosomes were originally thought to be garbage bags that can enable cells to remove useless junk, mounting evidence illustrates the nonnegligible role of exosomes in contributing to the pathological processes of various diseases 28,29, and kidney disease is no exception 30. A previous study demonstrated that exosomes secreted by damaged kidney cells can be transported to other normal kidney cells and activate fibroblasts, which promotes renal fibrosis and leads to a vicious cycle of renal injury 31.

Despite the inescapable role of exosomes in various kidney diseases, the exact upstream molecular regulation of exosome secretion remains unknown. TGF-β1 has been considered to be the most important fibrotic inducer in obstructed kidneys and can be released by all kinds of renal intrinsic cells and infiltrating inflammatory cells 27. In turn, TGF-β1 promotes a phenotypic response of tubular epithelial cells either to undergo apoptosis or to undergo epithelial-mesenchymal transition (EMT) 32. Recent studies indicate that TGF-β1 also plays a role in affecting the secretion and internal packages of exosomes in different tissues and cells 33,34. In our study, *in vivo* experiments indicated that TGF-β1 expression was positively correlated with exosome secretion and renal fibrosis (Figure 1). Subsequent *in vitro* experiments also confirmed that TGF-β1 promoted the secretion of tubular epithelial cell-delivered exosomes and further activated fibroblasts (Figure 2). Our findings demonstrate the important role of increased TGF-β1 in promoting exosome secretion of tubular epithelial cells in ureter obstructed kidneys.

Another consequential finding in our study is the upregulation of tubular cell-derived exosomes and the fibrotic role of exosomes on fibroblasts. Immunofluorescence staining of renal tissue sections confirmed that exosomes are predominantly localized in the renal proximal tubular epithelium, suggesting that proximal tubules are a major source of exosomes in ureteral obstruction-induced kidney injury (Figure 1). Similar results were observed in cell experiments; NRK-52E cells treated with TGF-β1 promoted the secretion of exosomes, which could be taken up by NRK-49F (Figure 2). In addition, these exosomes were able to promote fibroblast activation and aggravate kidney fibrosis both *in vitro* and *in vivo* (Figures 2 and 3), which manifests in the cell proliferation and production of α-SMA, collagen I and fibronectin 35. The RAB GTPase family regulates many steps in intracellular vesicular trafficking, such as budding, movement, tethering, docking and fusion 36. As a RAB GTPase family member, Rab27a is closely linked to the synthesis and secretion of exosomes 37. To further elucidate the specific role of exosomes in activating fibroblasts, Rab27a knockout mice and the exosome secretion inhibitor GW4869 38 were used to elaborate upon our findings (Figures 2 and 4). Our data underline an essential role for exosome-mediated epithelial-fibroblast communication in the pathogenesis of renal fibrosis induced by ureter obstruction.

Exosomes always contain large amounts of miRNAs and transfer these miRNAs to recipient cells, as inhibitors of target gene expression result in translational repression and mRNA degradation 39. GC cell-derived exosomal miR-23a can promote angiogenesis and provide a blood supply for the growth of GC cells by targeting PTEN 40. BMMSC-derived exosomal miR-144 led to restrained NSCLC cell proliferation and colony formation by downregulating CCNE1 and CCNE2 41. The miRNA-seq in our study showed that miR-21 was enriched in TGF-β1-stimulated tubular cell-delivered exosomes. MiR-21 is one of the most well-studied miRNAs and has been reported to participate in regulating fibroblast proliferation 42,43. The evidence points to the possibility that tubular cell-delivered exosomal miR-21 may activate fibroblasts, and our hypotheses were confirmed *in vitro* by cell transfection (Figure 5). The possible mRNA targets of miR-21 were predicted by TargetScan. Among all candidate mRNAs, PTEN has been studied in the context of fibroblast activation, and downregulation of PTEN is always affiliated with fibroblast activation and tissue fibrosis 44,45. A dual-luciferase assay showed that miR-21 can bind to the predicted binding site in the PTEN 3'UTR (Figure 6). In addition, an additional si-PTEN group *in vitro* and an anti-miR-21 LNA group *in vivo* were established, and PTEN/Akt pathway proteins were detected to further verify the miR-21/PTEN/Akt pathway (Figures 7 and 8). Taken together, the results arguably showed that tubule cells with exosomal miR-21 activated fibroblasts through the PTEN/Akt pathway in UUO kidneys.

It is important to note that we are not the first to study miR-21 in UUO induced renal fibrosis, several previous studies had already demonstrated that miR-21 plays important roles in promoting renal fibrosis in UUO models 46-49. What's more, some studies had even confirmed that miR-21/PTEN/Akt pathway played key roles in regulating UUO induced renal fibrosis 50. In spite of this, our study is still of great significance because previous studies had just focused on miR-21 levels in renal tissues but not exosomal miR-21. The role of exosomal miR-21 in UUO kidneys is rarely studied. Two recent studies emphasised that microvesicle-mediated delivery of miR-21 from injured epithelial cells could promote EMT of normal epithelial cells through PTEN and Akt pathway. However, the mechanisms were not deeply explored, there was no intervention with exosomal miR-21 and they did not delve into the mechanism of exosome secretion. More importantly, their studies were about microvesicle mediated cell communication among tubular epithelial cells, and our study focus on exosome mediated epithelial-fibroblast communication 23,51. In this way, our study is still of great novelty and significance. Furthermore, several studies have implied the specific roles of exosomal mRNA in regulating disease 52,53. In this regard, we have insufficient evidence to exclude the possibility that other components in exosomes may also play roles in mediating kidney fibrosis.

In conclusion, we demonstrated that tubular cell-derived exosomes play an important role in ureteral obstruction-induced hydronephrosis and long-term renal fibrosis. We found that the production of exosomes containing miR-21 increased after UUO. The delivery of tubular cell-derived exosomal miR-21 promoted fibroblast activation by targeting PTEN and influenced the PTEN/Akt pathway. These results introduce a new mechanism explaining how cell-cell communication is mediated by exosomes in UUO models and suggest that exosomes could be a new area for exploitation to retard or slow down the progression of renal fibrosis.
